# Draft Genome Sequence of *Pedococcus* sp. Strain 5OH_020, Isolated from California Grassland Soil

**DOI:** 10.1128/mra.00025-23

**Published:** 2023-05-08

**Authors:** Myka A. Green, Zoila I. Alvarez-Aponte, Valentine V. Trotter, Michiko E. Taga

**Affiliations:** a Department of Plant and Microbial Biology, University of California Berkeley, Berkeley, California, USA; b Environmental Genomics and Systems Biology Division, Lawrence Berkeley National Laboratory, Berkeley, California, USA; Indiana University, Bloomington

## Abstract

The draft genome sequence of the soil bacterium *Pedococcus* sp. strain 5OH_020, isolated on a natural cobalamin analog, comprises 4.4 Mbp, with 4,108 protein-coding genes. Its genome encodes cobalamin-dependent enzymes, including methionine synthase and class II ribonucleotide reductase. Taxonomic analysis suggests that it is a novel species within the genus *Pedococcus*.

## ANNOUNCEMENT

*Pedococcus* sp. strain 5OH_020 was isolated from ungrazed grassland topsoil at the Hopland Research and Extension Center (39.00 N, 123.08 W) by limiting dilution (1) in methionine-dropout VL60 medium containing 0.1 g/L each of xylan, glucose, xylose, and *N*-acetyl glucosamine (2) amended with 10 nM 5-hydroxybenzimidazolyl cobamide ([5-OHBza]Cba) that we synthesized according to reference 3. [5-OHBza]Cba is a commercially unavailable cobamide cofactor typically biosynthesized by methanogens and found in diverse environments, such as animal guts and contaminated groundwater (4–6). To our knowledge, this is the first report of a bacterium isolated on a cobamide other than vitamin B_12_ (cobalamin).

**FIG 1:**
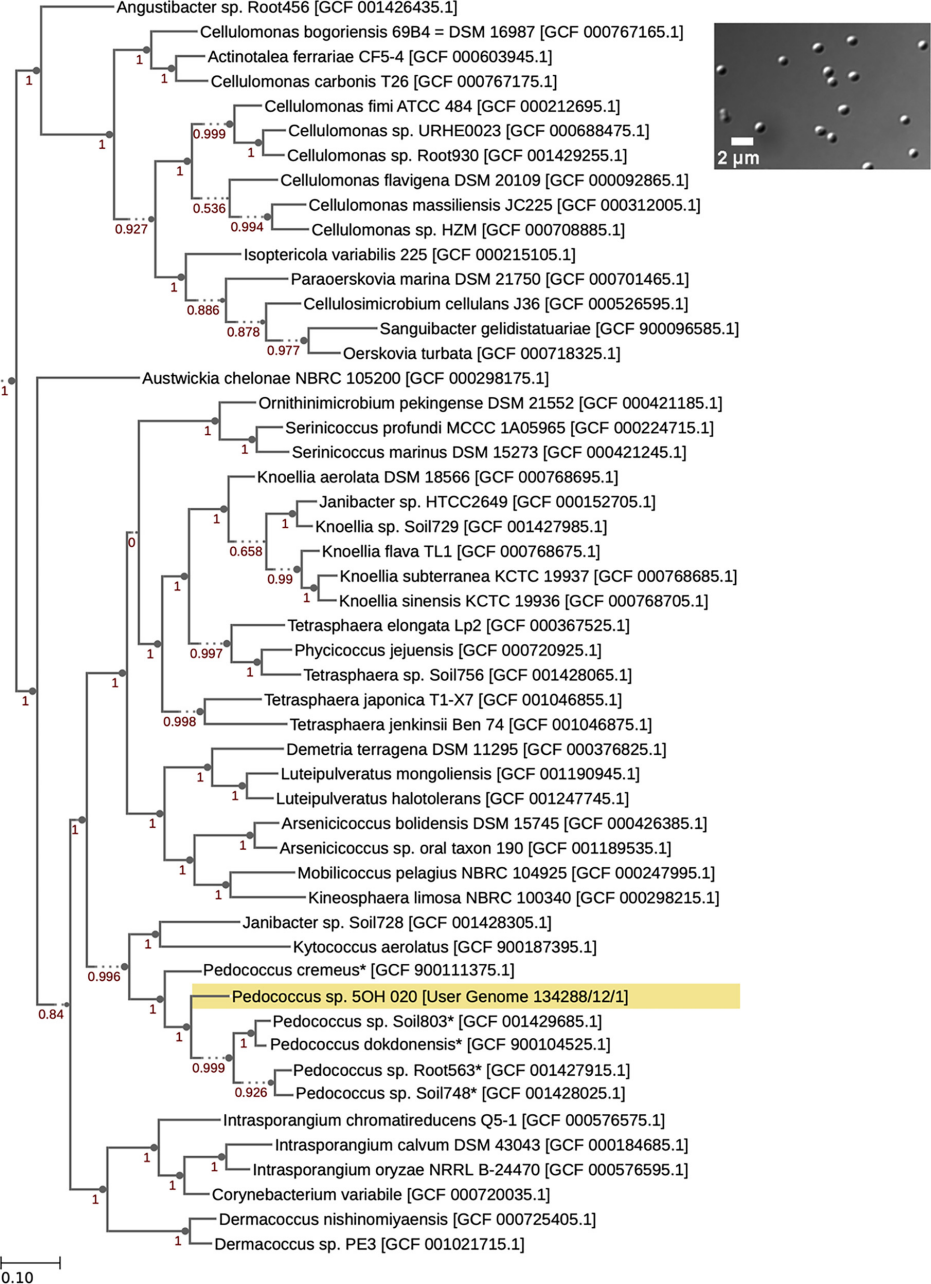
Morphology and phylogeny of *Pedococcus* sp. 5OH_020. A phylogenetic tree was generated using the Insert Genome into Species Tree v2.2.0 (19) application, which uses the FastTree phylogenetic inference method. Parameters were set to include 50 neighboring species representative of the class *Actinomycetia*. Strain 5OH_020 is highlighted in yellow. Asterisks indicate that the genus name *Phycicoccus* was changed to *Pedococcus* to match current taxonomic classifications according to the NCBI database. Numbers in red represent bootstrap values (1,000 replicates). (Inset) Differential interference contrast (DIC) microscopy of *Pedococcus* sp. 5OH_020 cells at ×100 magnification using a Zeiss AxioImager DIF M1 microscope. Image captured using a Hamamatsu Orca 03 camera.

Strain 5OH_020 was cultured at 28°C in the isolation medium, or on agar plates with the same medium at 2× concentration. The isolate is aerobic, Gram positive (7), and cocci-shaped (Fig. 1, inset), forming circular, cream-colored colonies after 12 days and growing only when [5-OHBza]Cba or cobalamin is provided. We Sanger sequenced 16S rRNA amplicons using the primers 27F and 1492R. After noting the isolate’s low similarity to its closest NCBI BLAST database pairwise matches (<98.65%) (8), we performed whole-genome sequencing.

DNA was extracted from a pure liquid culture using the DNeasy blood and tissue kit (Qiagen) protocol for Gram-positive bacteria. Subsequent library preparation and sequencing steps were carried out by Novogene Bioinformatics Technology Co., Ltd. (Beijing, China). Library construction was performed using the NEBNext DNA library prep kit, following the manufacturer’s instructions. After end repair, dA-tailing, and further ligation with NEBNext adapters, the DNA was sequenced using the NovaSeq 6000 platform (Illumina Inc., San Diego, CA, USA) to generate 150-bp paired-end reads, yielding 9,600,674 total reads after a raw data filtering step using FASTP v0.32.2 (9).

Data processing and analysis were performed using the Department of Energy Systems Biology Knowledgebase (10). The raw reads were trimmed and quality controlled using Trimmomatic v0.36 (11). The genome was assembled *de novo* via SPAdes v3.15.3 (12) and quality checked using QUAST v4.4 (13), generating the assembly statistics in Table 1. The genome was annotated and reannotated using Prokka v.1.14.5 (14) and distilled using DRAM v0.1.2 (15). The 4.4-Mbp genome was quality checked using CheckM v1.0.18 (16). Default parameters were used for all software.

**TABLE 1:** Assembly statistics generated using QUAST v4.4

Assembly statistic	Result
No. of contigs	33
Largest contig (bp)	783,609
Total length (bp)	4,427,096
Coverage (×)	325
*N*_50_ (bp)	428,675
*N*_75_ (bp)	278,038
*L*_50_	5
*L*_75_	8
G+C content (%)	69.75

The genome was classified as belonging to the genus *Pedococcus*, with no further classification at the species level using GTDB-Tk v1.7.0 (17). The amino acid identity with *Pedococcus* sp. strain Soil748 was 95.61%. Using FastANI v1.33, an average nucleotide identity of 81.17% was indicated with the genus type strain Pedococcus dokdonensis (18)*. Pedococcus* sp. 5OH_020 forms an independent branch within the *Pedococcus* clade (Fig. 1) (19).

*Pedococcus* sp. 5OH_020 belongs to the phylum *Actinobacteria*, which is renowned for its production of clinically useful natural products (20). Among the 4,108 annotated protein-coding genes, two natural product biosynthetic clusters were detected using antiSMASH v6.0 (21). The genome also encodes enzymes predicted to degrade arabinan, xyloglucan, and amorphous cellulose, suggesting that strain 5OH_020 may be capable of degrading plant matter in soil.

The genome encodes two cobalamin-dependent enzymes: methionine synthase and class II ribonucleotide reductase. No cobamide biosynthesis genes were present, consistent with the cobamide dependence observed in culture. Thus, strain 5OH_020 likely relies on other microbes in the soil for cobamides.

### Data availability.

The draft genome sequence of *Pedococcus* sp. 5OH_020 was deposited at DDBJ/ENA/GenBank under the accession number JAPYZV000000000. The version described in this paper is version JAPYZV010000000. The NCBI SRA accession number for the raw reads is SRR22578002. The BioProject and BioSample accession numbers for this project are PRJNA891762 and SAMN31357841, respectively.
